# Comparison of Early Functional Recovery Following Triportal Robot‐Assisted and Uniportal Video‐Assisted Segmentectomy in Patients With Early‐Stage Non‐Small Cell Lung Cancer: A Propensity Score‐Matched Analysis

**DOI:** 10.1111/1759-7714.70041

**Published:** 2025-03-12

**Authors:** Yan‐Cheng Wang, Hai‐xiao Diao, Lin Xu, Zhong‐Min Peng

**Affiliations:** ^1^ Department of Thoracic Surgery, Shandong Provincial Hospital Affiliated to Shandong First Medical University Shandong First Medical University Jinan P. R. China; ^2^ National Clinical Research Center for Cancer Chinese Academy of Medical Sciences and Peking Union Medical College Beijing P. R. China

**Keywords:** non‐small cell lung cancer, postoperative recovery, robot‐assisted thoracoscopic surgery, segmentectomy, uniportal video‐assisted thoracic surgery

## Abstract

**Background:**

Robot‐assisted thoracoscopic surgery (RATS) is more precise and flexible than video‐assisted thoracoscopic surgery (VATS) for early‐stage non‐small cell lung cancer (NSCLC) treatment. This study compared the early postoperative functional recovery of patients who underwent triportal RATS with that of patients who underwent uniportal video‐assisted thoracic surgery (UVATS) for segmentectomy.

**Methods:**

This observational, prospective study included 172 patients with clinical stage I or II peripheral NSCLC who underwent RATS or UVATS segmentectomy. Propensity score matching (PSM) was used to balance differences between groups. The functional recovery data were collected during the first 4 weeks after discharge via portable devices and questionnaires (EORTC QLQ‐C30, Christensen Fatigue Scale, MD Anderson Symptom Inventory, and Leicester Cough Questionnaire).

**Results:**

After PSM, the baseline characteristics were consistent between the groups. RATS was associated with shorter operation time and lower total drainage volume compared to UVATS. However, RATS was associated with more cases of severe postoperative pain. Despite this, patients who underwent RATS recovered well, showed good short‐term outcomes in fatigue and physical function, and experienced few postoperative adverse events. The differences in average daily step count and sleep duration were not significant. In terms of global health status (GHS), RATS was slightly but nonsignificantly advantageous.

**Conclusions:**

In the enhanced recovery after surgery (ERAS) pathway, triportal RATS has potential benefits in terms of perioperative and early postoperative functional recovery after segmentectomy.

**Trial Registration:**

Biomedical Research Ethics Committee of Shandong Province: 2022‐580; Chinese Clinical Trial Registry: ChiCTR2300067977

## Graphical abstract

This study combined subjective and objective data to analyze the differences in early postoperative functional recovery (within 4 weeks) between triportal RATS segmentectomy and uniportal VATS segmentectomy. Our findings suggest that RATS has potential advantages in early postoperative functional recovery and provides a viable alternative for surgical decision‐making in early‐stage NSCLC.

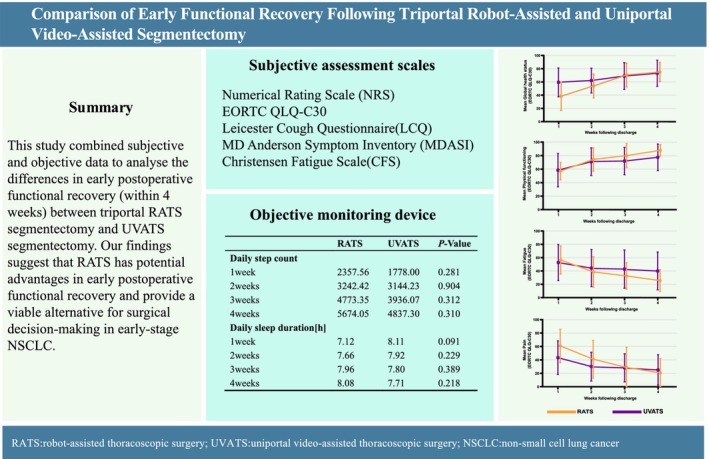

**Abbreviations**
EORTC QLQ‐C30: European organization for research and treatment of cancer quality of life questionnaire‐core 30
ERAS: enhanced recovery after surgery
GHS: global health status
MDASI: MD anderson symptom inventory
MIS: minimally invasive surgery
NSCLC: non‐small cell lung cancer
PSM: propensity score matching
QOL: quality of life
RATS: robot‐assisted thoracoscopic surgery
UVATS: uniportal video‐assisted thoracoscopic surgery
VATS: video‐assisted thoracoscopic surgery

## 1 Introduction

Lung cancer is a major global health concern. Non‐small cell lung cancer (NSCLC) accounts for 85% of all cases [1]. Universalization via high‐resolution computed tomography (CT) has significantly increased the detection rate of small lung nodules [2, 3]. Surgical resection with lymph node dissection is the preferred treatment for early‐stage NSCLC [4, 5]. Segmentectomy has been proven to be an alternative to lobectomy for the treatment of early‐stage NSCLC, as it preserves more lung tissue with fewer complications and faster recovery [6, 7].

Minimally invasive surgery (MIS) is now the standard treatment for early‐stage NSCLC, enhancing the safety and precision of segmentectomy and supporting enhanced recovery after surgery (ERAS) protocols [8, 9]. Compared to traditional triportal video‐assisted thoracoscopic surgery (VATS), uniportal video‐assisted thoracoscopic surgery (UVATS) has become increasingly established as a mainstream approach in thoracic surgery due to its reduced surgical access trauma [10, 11]. The uptake of UVATS in China has progressed more rapidly, becoming the preferred approach in many centres [12]. Compared with VATS, robot‐assisted thoracoscopic surgery (RATS) allows high‐resolution, three‐dimensional (3D) visibility, utilizes mechanical arms with multiple degrees of freedom, and has excellent operational flexibility [13]. Consequently, RATS is increasingly favored for the treatment of NSCLC [14]. Choosing the optimal surgical approach is crucial for patients to experience favorable outcomes and satisfactory recovery. Although numerous studies have revealed differences between RATS and VATS in lobectomy, few have investigated segmentectomy. Yang et al. reported that RATS was superior to VATS for segmentectomy in operation time, blood loss, hospital stay, and analgesic medication usage [15]. Zhou et al. reported no significant differences in 5‐year recurrence‐free survival (RFS) or overall survival (OS) between patients who received RATS and VATS segmentectomy [16]. Research on rehabilitation after discharge remains scarce, and the differences between the two approaches are still unclear.

This study aimed to comprehensively compare early functional recovery in early‐stage NSCLC patients following triportal RATS and UVATS segmentectomy. We collected subjective data from patients, such as pain, fatigue, cough, and quality of life (QOL) scores through questionnaires, and objective data on daily step count and sleep duration via a portable digital device. By combining subjective and objective data, we analyzed the potential differences between the two surgical approaches across symptoms, functions, and QOL.

## 2 Study Design and Methods

### 2.1 Study Design and Patient Characteristics

This observational, prospective study included 172 patients with suspected or diagnosed NSCLC (clinical stages I and II) treated at the Thoracic Surgery Department of Shandong Provincial Hospital between September 2022 and March 2024.

The inclusion criteria were as follows: (1) consent for RATS or UVATS segmentectomy, (2) tumors located in the outer third of the lung with more than 50% GGO and tumors smaller than 2 cm if the solid component exceeded 50% [5, 17], (3) clinical stage N0 [17], (4) no preoperative radiotherapy, chemotherapy, or other treatments. The exclusion criteria were (1) limited activity or psychiatric disorders, (2) prior lung surgery or concurrent malignancies, or (3) neoadjuvant therapy.

Patients self‐selected either RATS or UVATS and were grouped accordingly. Baseline characteristics and perioperative outcomes were recorded: age, gender, body mass index (BMI), smoking status, maximum voluntary ventilation (MVV), forced expiratory volume in 1 s (FEV1), single‐breath diffusing capacity of the lung for carbon monoxide (DLCO‐SB), Charlson comorbidity index (CCI) [18], GGO components, American Society of Anesthesiologists Physical Status (ASA‐PS), clinical TNM stage, pathological T stage [19], distribution of resected segments, pathology type, tumor diameter, lymph node resection number, operation time, total drainage volume, chest drain duration, and length of hospital stay. The primary endpoint of this study is the difference in early postoperative functional recovery (4 weeks after discharge) between triportal RATS and UVATS segmentectomy. The secondary endpoints are the perioperative outcome differences between the two surgical approaches, with the aim of identifying the superior surgical method.

This study was conducted in accordance with the Declaration of Helsinki. The participants were informed of the study's purpose and procedures before surgery and signed informed consent forms. The study was also approved by the Ethics Committee on Biomedical Research of Shandong Provincial Hospital (NO. 2022‐580).

### 2.2 Surgical Techniques and Clinical Management

Preoperative preparation including individualized assessment of cardiopulmonary function (pulmonary function tests, electrocardiogram, cardiac ultrasound and coronary CT if necessary); clarification of clinical TNM staging and exclusion of imaging metastasis (abdominal and adrenal ultrasound, chest CT, brain CT). A multidisciplinary consultation with oncologists, thoracic surgeons, and pulmonologists was conducted. The ERAS program, which is based on current guidelines, was the standard management protocol in our department [20].

In this study, each surgery was performed jointly by two surgeons (the lead surgeon and the first assistant), with the lead surgeon being the same senior thoracic surgeon with more than 10 years of experience in minimally invasive thoracic surgery. Additionally, RATS and UVATS procedures were alternately performed to ensure consistency in surgical technique. Operations were performed under standard general anesthesia in the lateral decubitus position, and all patients underwent anatomical segmentectomy. For triportal UVATS, an incision about 4 cm was made at the fourth or fifth intercostal space between the midaxillary and anterior axillary lines. For RATS, the da Vinci Si system (Intuitive Surgical, Sunnyvale, California) was used, employing a three‐arm technique modified from Dylewski [21]. A 4 cm incision at the fifth intercostal space along the anterior axillary line served as the utility port and first instrument port (#1 arm). The lens port and second instrument port (#2 arm) were positioned at the eighth intercostal space along the mid‐axillary and posterior axillary lines, each approximately 1 cm in size. An 8 cm distance between ports, without rib spreading. Intersegmental planes were identified via the inflation‐deflation method, which involves ligating or cutting the target segmental arteries and veins [22]. N1 and N2 lymph nodes were sampled when feasible according to the National Comprehensive Cancer Network (NCCN) guidelines [5]. In some cases, only N1 nodes were sampled due to congenital anomalies or exposure issues. A 24FR drainage tube was placed dorsally via the UVATS incision and through the lens port in RATS.

All patients received standardized postoperative pain management during hospitalization, which included scheduled administration of nonsteroidal anti‐inflammatory drugs (NSAIDs) and opioids as needed. No analgesic medications were prescribed upon discharge to ensure that the follow‐up assessment results were not disturbed by differences in analgesic use. Low‐molecular‐weight heparin was routinely used for thromboprophylaxis. On the first postoperative day, all patients underwent chest digital radiography (DR) to assess lung re‐expansion and effusion. The drainage tube was removed if there was no air leakage and if the drainage volume was less than 200 mL within 24 h. Patients without complications were discharged 1 to 2 days after tube removal. All patients were discharged successfully and did not receive any adjuvant treatment within 4 weeks after discharge.

### 2.3 Follow‐Up Methods and Assessment Scales

We collected early postoperative recovery data from patients at 4 weeks after discharge. The questionnaires were sent to patients via WeChat on Days 7, 14, 21, and 28 after discharge. Patients who did not respond within 24 h were reminded, and those who did not respond within 48 h were considered lost to follow‐up. Daily step counts and sleep duration were monitored via smart bands (Mi Band 6 [23, 24], Xiaomi Corporation, Beijing, China), and the data were processed via “Xiaomi Mi Fit” software to generate reports on average daily steps and sleep duration. All assessment scales are shown below.

#### 2.3.1 Numerical Rating Scale (NRS) [25]

A common scale for assessing patients' subjective pain, ranging from 0 (*no pain*) to 10 (*severe pain*).

#### 2.3.2 EORTC QLQ‐C30 [26]

A standardized questionnaire for assessing QOL in cancer patients. It consists of 30 items divided into three scales: global health status (GHS), functional scales, and symptom scales. Higher scores on the functional and GHS scales indicate better functional recovery and QOL, whereas higher scores on the symptom scales indicate worse adverse reactions.

#### 2.3.3 Leicester Cough Questionnaire(LCQ) [27]

Evaluates the impact of chronic cough on QOL. It contains 19 items across three dimensions: physical, psychological, and social, with scores ranging from 1 to 7. Higher scores indicate better recovery.

#### 2.3.4 MD Anderson Symptom Inventory (MDASI) [28]

Measures symptom severity and its impact on daily life in cancer patients. It includes 13 core symptom items and 6 interference items. Each item is rated from 0 (*no symptoms*) to 10 (*most severe symptoms*).

#### 2.3.5 Christensen Fatigue Scale (CFS) [29]

A single‐item self‐assessment scale for fatigue, scored from 1 to 10, with higher scores indicating greater fatigue.

### 2.4 Statistical Analysis

To mitigate the impact of nonrandom patient allocation and confounding variables, we employed a propensity score‐matching (PSM) analysis including age, gender, BMI, MVV, FEV1, DLCO‐SB, smoking status, CCI, ASA‐PS, and tumor diameter. The match tolerance was 0.02.

All statistical analyses were performed with IBM SPSS 27.0 (IBM Corp, Armonk, NY, USA). Normally distributed continuous variables were analyzed via Student's t‐test and are reported as mean ± SD. Nonnormally distributed data were analyzed with the Mann–Whitney U test and are presented as medians and interquartile ranges (IQR). A chi‐square test was used for dichotomous variables. A two‐tailed *p*‐value < 0.05 was considered significant.

## 3 Results

### 3.1 Patient Involvement and Baseline Characteristics

**FIGURE 1:**
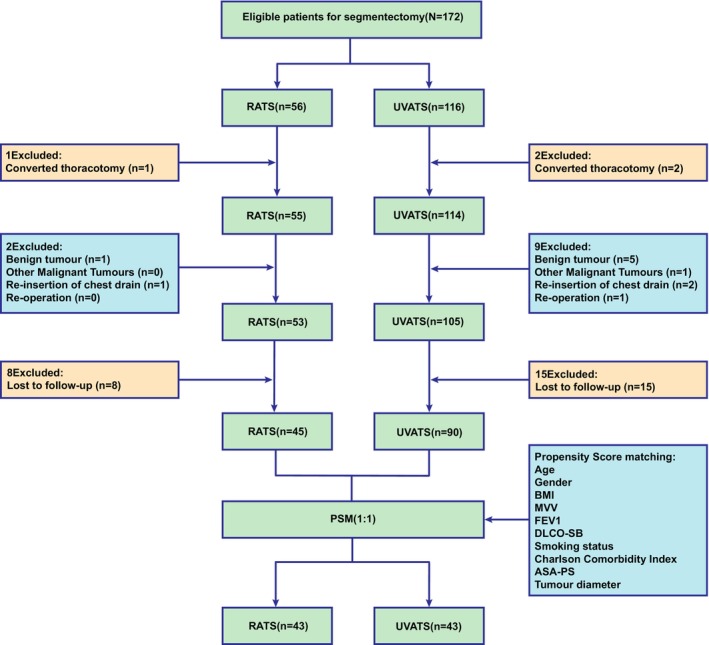
Flow chart of patient enrollment. PSM, propensity score matching; RATS, robot‐assisted thoracic surgery; UVATS, uniportal video‐assisted thoracic surgery.

The patient inclusion process is illustrated in Figure 1. From September 2022 to March 2024, 172 patients (56 RATS, 116 UVATS) were initially screened for surgery eligibility. Fourteen patients were excluded for various reasons, including conversion to thoracotomy (3 patients), benign tumors and other malignancies (7 patients), reinsertion of a chest drain (3 patients), and reoperation (1 patient). Ultimately, 158 patients underwent the planned surgery (53 RATS, 105 UVATS) and were discharged. All patients received the follow‐up questionnaire. A total of 135 patients (45 RATS, 90 UVATS) completed the required 4‐week follow‐up. After PSM, 43 pairs (86 patients) were created. The baseline characteristics of the two groups were well balanced (all *p* > 0.05).

### 3.2 Perioperative Outcomes

Compared with UVATS, RATS had a shorter operation time [100 (90–115) min vs. 110 (95–130) min, *p* = 0.012] and a lower total drainage volume [250 (180–330) mL vs. 300 (210–410) mL, *p* = 0.046] (Table 1). No significant differences were found in lymphadenectomy (*p* = 0.401), duration of chest drainage (*p* = 0.373), length of stay (*p* = 0.245), or distribution of resected segments (*p* = 0.999, Table S1).

**TABLE 1:** Patient clinicopathological characteristics and perioperative outcomes before and after propensity score matching (PSM).

Characteristics	Before PSM (*n* = 135)			After PSM (*n* = 86)		
	RATS (*n* = 45)	UVATS (*n* = 90)	*p*	RATS (*n* = 43)	UVATS (*n* = 43)	*p*
Age [years], mean ± SD	57.67 ± 7.76	55.58 ± 11.01	0.257	57.19 ± 7.60	57.51 ± 10.32	0.868
Gender, male, *n* (%)	18 (36.00)	27 (31.76)	0.614	16 (37.20)	15 (34.88)	0.822
BMI [kg/m^2^], mean ± SD	24.54 ± 3.49	24.93 ± 3.51	0.539	24.64 ± 3.53	24.20 ± 3.49	0.565
Smoking status, *n* (%)	6 (13.33)	15 (16.67)	0.614	6 (13.95)	6 (13.95)	1.000
FEV1 (%), mean ± SD	98.82 ± 15.15	97.53 ± 15.81	0.652	98.39 ± 15.37	98.07 ± 16.95	0.926
MVV (%), mean ± SD	89.73 ± 17.41	91.25 ± 19.76	0.662	89.53 ± 17.77	86.44 ± 16.89	0.411
DLCO‐SB (%), mean ± SD	90.22 ± 15.41	89.80 ± 13.85	0.871	90.32 ± 15.70	89.41 ± 12.73	0.769
Charlson Comorbidity Index, median (IQR)	2.00 (2.00–3.00)	2.00 (2.00–3.00)	0.522	2.00 (2.00–3.00)	2.00 (2.00–3.00)	0.692
GGO, *n* (%)	37 (82.22)	79 (87.78)	0.382	35 (81.40)	39 (90.70)	0.213
ASA‐PS, *n* (%)			0.495			0.676
1–2	43 (95.56)	82 (91.11)		41 (95.35)	39 (90.70)	
3–4	2 (4.44)	8 (8.89)		2 (4.65)	4 (9.30)	
Clinical TNM stage, *n* (%)			0.776			0.662
IA1	15 (33.3)	22 (24.4)		15 (34.9)	9 (20.9)	
IA2	23 (51.1)	53 (58.9)		21 (48.8)	24 (55.8)	
IA3	5 (11.1)	12 (13.3)		5 (11.6)	8 (18.6)	
IB	1 (2.2)	2 (2.2)		1 (2.3)	1 (2.3)	
IIA	0	0		0	0	
IIB	1 (2.2)	1 (1.1)		1 (2.3)	1 (2.3)	
IIIA	0	0		0	0	
Pathologic T stage, *n* (%)			0.704			0.642
Tis	6 (13.3)	23 (25.6)		6 (14.0)	11 (25.6)	
T1mi	13 (28.9)	22 (24.4)		12 (27.9)	9 (20.9)	
T1a	4 (8.9)	7 (7.8)		4 (9.3)	4 (9.3)	
T1b	18 (40.0)	30 (33.3)		17 (39.5)	13 (30.2)	
T1c	4 (8.9)	6 (6.7)		4 (9.3)	5 (11.6)	
T2a	0	1 (1.1)		0	0	
T2b	0	0		0	0	
T3	0	1 (1.1)		0	1 (2.3)	
T4	0	0		0	0	
Pathology, *n* (%)			1.000			1.000
Adenocarcinoma, *n* (%)	43 (95.6)	85 (94.4)		41 (95.3)	42 (97.7)	
Squamous, *n* (%)	2 (4.4)	5 (5.6)		2 (4.7)	1 (2.3)	
Diameter of tumor [cm], median (IQR)	1.2 (1.0–1.65)	1.2 (0.8–1.6)	0.426	1.2 (1.0–1.6)	1.4 (0.9–1.8)	0.798
Lymphadenectomy, median (IQR)	5 (4–6)	5 (5–7)	0.409	5 (4–6)	6 (4–7)	0.401
Operation time [min], median (IQR)	100 (90–115)	112.5 (90–136.25)	**0.002**	100 (90–115)	110 (95–130)	**0.012**
Total drainage volume [mL], median (IQR)	250 (157.5–357.5)	250 (157.5–357.5)	**0.026**	250 (180–330)	300 (210–410)	**0.046**
Duration of chest drainage [d], median (IQR)	2 (2–3)	2 (2–3)	0.812	2 (2–3)	2 (2–3)	0.373
Length of stay [d], median (IQR)	3 (2–3)	3 (3–4)	0.214	3 (2–3)	3 (3–3)	0.245

*Note:* Bold text hinted that these variables were statistically significant.

Abbreviations: ASA‐PS, American society of anesthesiologists physical status classification system; BMI, body mass index; DLCO‐SB, single‐breath diffusing capacity of the lung for carbon monoxide; FEV1, forced expiratory volume in 1 s; GGO, ground‐glass opacity; IQR, interquartile range; MVV, maximum voluntary ventilation; PSM, propensity score matching; SD, standard deviation.

### 3.3 Exercise and Sleep Indicators

There were no significant differences in the daily step count (Week 1, *p* = 0.281; Week 2, *p* = 0.904; Week 3, *p* = 0.312; Week 4, *p* = 0.310) or daily sleep duration (Week 1, *p* = 0.091; Week 2, *p* = 0.229; Week 3, *p* = 0.389; Week 4, *p* = 0.218) between the groups. However, the daily step count for RATS was consistently higher than that for UVATS on average (Table 2).

**TABLE 2:** Weekly average daily step count and sleep duration after discharge.

	After PSM (*n* = 86)		
	RATS (*n* = 43), mean	UVATS (*n* = 43), mean	*p*
Daily step count			
1 week	2357.56	1778.00	0.281
2 weeks	3242.42	3144.23	0.904
3 weeks	4773.35	3936.07	0.312
4 weeks	5674.05	4837.30	0.310
Daily sleep duration [h]			
1 week	7.12	8.11	0.091
2 weeks	7.66	7.92	0.229
3 weeks	7.96	7.80	0.389
4 weeks	8.08	7.71	0.218

### 3.4 Assessment Scales

#### 3.4.1 EORTC QLQ‐C30

**FIGURE 2:**
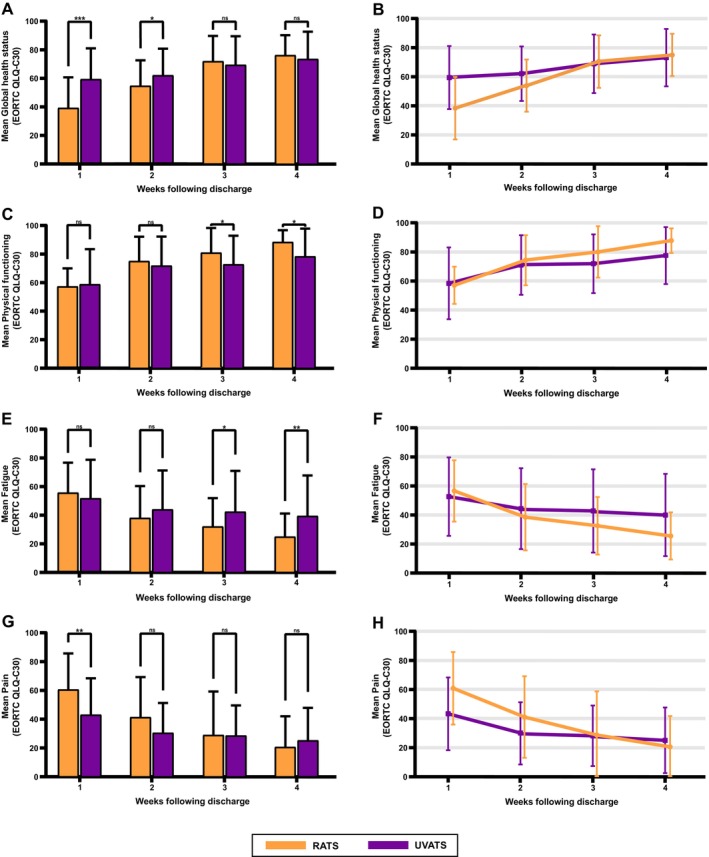
Comparison of scores and trends in general health, physical functioning, fatigue, and pain on the EORTC QLQ‐C30 questionnaire from 1 to 4 weeks after discharge. (A) and (B), Comparison of Global Health Status scores and trends. (C) and (D), Comparison of physical functioning scores and trends. (E) and (F), Comparison of fatigue scores and trends. (G) and (H), Comparison of pain scores and trends. RATS, robot‐assisted thoracic surgery; UVATS, uniportal video‐assisted thoracic surgery. (Significant symbol: ns, *p* ≥ 0.05; *, *p* < 0.05; **, *p* < 0.01; ***, *p* < 0.001).

In the first week after discharge, RATS reported lower GHS scores (39.15 vs. 59.30, *p* < 0.001) and higher pain scores (60.47 vs. 43.02, *p* = 0.002) than UVATS (Table 3). In the second week, RATS presented pain alleviation (*p* = 0.095), but the GHS score (54.65 vs. 62.02, *p* = 0.029) remained lower. In week three, RATS presented better physical functioning (80.16 vs. 72.25, *p* = 0.040), fatigue (29.19 vs. 41.86, *p* = 0.043), insomnia (13.95 vs. 31.78, *p* = 0.013), and appetite loss (10.85 vs. 24.03, *p* = 0.017). By week four, RATS presented significant advantages in physical functioning (87.91 vs. 77.83, *p* = 0.028), appetite loss (9.30 vs. 23.26, *p* = 0.023), and fatigue (24.55 vs. 39.02, *p* = 0.009). No significant differences were noted in the other measures. Figure 2 illustrates the recovery trend throughout the follow‐up period. Initially, RATS presented lower GHSs and higher pain scores, but there were significant improvements over time, reaching recovery levels comparable to those in UVATS at the end of the follow‐up period (Figure 2A,G). In terms of physical functioning and fatigue (Figure 2C,E), no significant differences were observed during the first week; however, by week four, RATS had better scores than UVATS. Longitudinal analysis confirmed that RATS recovered faster and had better final outcomes than UVATS (Figure 2B,D,F,H).

**TABLE 3:** EORTC QLQ‐C30 data (4 weeks after discharge).

	After PSM (*n* = 86)		
	RATS (*n* = 43)	UVATS (*n* = 43)	*p*
1 week			
Global health status (mean)	39.15	59.30	**< 0.001**
Functional scales (mean)			
Physical functioning	57.21	58.76	0.295
Role functioning	50.00	58.14	0.126
Emotional functioning	75.58	75.78	0.979
Cognitive functioning	82.56	75.58	0.413
Social functioning	63.18	65.50	0.737
Symptom scales/items(mean)			
Fatigue	55.56	51.68	0.363
Nausea and vomiting	15.12	18.99	0.444
Pain	60.47	43.02	**0.002**
Dyspnea	47.29	48.06	0.946
Insomnia	26.36	38.76	0.092
Appetite loss	31.78	42.64	0.163
Constipation	25.58	23.26	0.565
Diarrhea	15.50	13.18	0.710
Financial difficulties(mean)	20.16	24.03	0.444
2 weeks			
Global health status (mean)	54.65	62.02	**0.029**
Functional scales (mean)			
Physical functioning	74.57	71.32	0.391
Role functioning	66.67	62.79	0.360
Emotional functioning	78.68	79.65	0.621
Cognitive functioning	85.66	77.52	0.071
Social functioning	67.83	69.38	0.971
Symptom scales/items(mean)			
Fatigue	37.47	43.41	0.420
Nausea and vomiting	6.98	11.24	0.556
Pain	40.69	29.84	0.095
Dyspnea	43.41	34.11	0.272
Insomnia	23.26	31.01	0.232
Appetite loss	19.38	28.68	0.308
Constipation	20.93	16.28	0.395
Diarrhea	10.08	9.30	0.686
Financial difficulties (mean)	19.38	24.81	0.323
3 weeks			
Global health status (mean)	71.32	68.80	0.464
Functional scales (mean)			
Physical functioning	80.16	72.25	**0.040**
Role functioning	72.09	60.85	0.111
Emotional functioning	80.81	78.10	0.677
Cognitive functioning	86.05	79.84	0.181
Social functioning	73.26	69.38	0.313
Symptom scales/items (mean)			
Fatigue	29.19	41.86	**0.043**
Nausea and vomiting	5.04	10.85	0.203
Pain	28.29	27.91	0.418
Dyspnea	32.56	32.56	0.825
Insomnia	13.95	31.78	**0.013**
Appetite loss	10.85	24.03	**0.017**
Constipation	10.08	14.73	0.676
Diarrhea	6.20	9.30	0.533
Financial difficulties (mean)	15.50	23.53	0.130
4 weeks			
Global health status (mean)	75.78	73.06	0.621
Functional scales (mean)			
Physical functioning	87.91	77.83	**0.028**
Role functioning	79.46	70.54	0.272
Emotional functioning	85.66	81.20	0.961
Cognitive functioning	88.76	82.56	0.187
Social functioning	79.84	70.93	0.100
Symptom scales/items (mean)			
Fatigue	24.55	39.02	**0.009**
Nausea and vomiting	4.26	7.36	0.709
Pain	20.16	24.81	0.320
Dyspnea	20.16	27.91	0.251
Insomnia	18.60	23.26	0.429
Appetite loss	9.30	23.26	**0.023**
Constipation	7.75	11.63	0.403
Diarrhea	6.98	10.08	0.541
Financial difficulties (mean)	14.73	23.26	0.238

*Note:* Bold text hinted that these variables were statistically significant.

#### 3.4.2 Leicester Cough Questionnaire

During the first and second weeks after discharge (Table S2), both groups had comparable scores in all dimensions (all *p* > 0.05). At week three, RATS had slightly better physical scores (5.45 vs. 5.13, *p* = 0.031). By week four, the difference between the two groups was not significant (all *p* > 0.05).

#### 3.4.3 MD Anderson Symptom Inventory

In the first week (Table S3), RATS reported higher pain scores (5.63 vs. 4.49, *p* = 0.007) but lower scores for nausea [1 (0–3) vs. 1 (1–4), *p* = 0.039] and difficulty remembering [2 (0–4) vs. 3 (2–5), *p* = 0.004]. There were no significant differences in other symptoms or daily life interference (all *p* > 0.05). During the second week, RATS maintained its advantages in nausea [1 (0–2) vs. 1 (1–3), *p* = 0.027] and difficulty remembering [2 (1–3) vs. 3 (2–4), *p* = 0.009] and experienced fewer sleep disturbances [1(0–4) vs. 3 (1–4), *p* = 0.020]. In week three, RATS scored lower in fatigue [3 (2–4) vs. 3 (2–5), *p* = 0.047], disturbed sleep [2 (0–2) vs. 2 (1–4), *p* = 0.036]. By week four, the trend towards functional recovery plateaued in both groups and was consistent. RATS demonstrated better performance in fatigue [2 (2, 3) vs. 3 (2–4), *p* = 0.043] and lack of appetite [2 (1, 2) vs. 2 (1–4), *p* = 0.037]. In other respects, the two groups were comparable.

#### 3.4.4 Christensen Fatigue Scale

Fatigue was not significantly different between the RATS and UVATS during 4 weeks (Week 1, *p* = 0.056; Week 2, *p* = 0.072; Week 3, *p* = 0.166; Week 4, *p* = 0.068). Both groups improved over time, with the RATS exhibiting a slightly better recovery trend (Table S4).

## 4 Discussion

With the implementation of ERAS in thoracic surgery, surgical protocols have become more precise. Segmentectomy has become a crucial surgical option for the treatment of early‐stage NSCLC. Zhang et al. revealed that RATS and VATS segmentectomy are comparable in safety and feasibility [30]. Pan et al. reported that while both techniques offer similar survival outcomes, RATS is superior in perioperative outcomes [31]. However, existing studies have focused primarily on comparing surgical outcomes and short‐term recovery during hospitalization between RATS and VATS segmentectomy. Therefore, it is necessary to compare early functional recovery after RATS and VATS segmentectomy within the ERAS protocol. Our findings indicate that although triportal RATS underperformed in the early postoperative period, most health indicators improve over time and eventually become comparable to or better than those of UVATS. It is important to note that the results observed in this study were obtained with the triportal RATS port placement. The impact of other incision placements (such as single‐port or four‐port) on perioperative outcomes and early postoperative functional recovery remains to be explored.

Xie et al. reported that, compared with VATS and open surgery, RATS has a shorter operation time and lower drainage volume during segmentectomy [32]. In our study, RATS was associated with a shorter operation time and lower total drainage volume compared to UVATS, with other perioperative outcomes being comparable, highlighting the efficiency and precision of RATS in segmentectomy. We attribute these advantages to the characteristic features of RATS: 3D visibility, flexible mechanical arms, and the EndoWrist system [33], which filters out hand tremors and enhances hand‐eye coordination. These enable more precise manipulation, effective bleeding control, and reduced tissue damage, thereby reducing fluid exudation and accelerating recovery. Additionally, RATS enables the surgeon to independently control the lens, effectively minimizing the impact of assistant‐related factors on operation time. Merritt et al. noted that experienced surgeons and well‐coordinated surgical teams also influence the operation time and total drainage volume [34].

The development of MIS has accelerated ERAS realization, making rapid postoperative recovery a critical factor in surgical decision‐making. Huang et al. demonstrated that pain and fatigue are the primary obstacles to early functional recovery after surgery [35]. The results from a randomized clinical trial by Jin et al. revealed that by the fourth postoperative week, the pain scores following RATS lobectomy were lower than those following VATS, and overall pain was comparable between the two groups [13]. Asemota et al. confirmed that compared with VATS and open surgery, RATS results in less fatigue [36]. In our study, the EORTC QLQ‐C30, MDASI, and NRS results indicated that the RATS experienced more pain during the first week after discharge. However, as patients recovered, RATS presented a better recovery trend than UVATS, with comparable pain scores. According to the EORTC QLQ‐C30 and MDASI, in fatigue recovery, RATS had significantly lower symptom scores than UVATS. Although the differences on the CFS scales were not statistically significant, subtle differences in recovery trends were still evident between the groups.

We consider that severe pain in the early period after triportal RATS may result from the three‐incision approach, which causes more damage to the intercostal muscles, nerves, and parietal pleura than does UVATS with a single incision. However, a single incision for all surgical instruments in UVATS may increase pressure and torsion on intercostal tissues. In contrast, the innovative wrist rotation system of RATS allows the robotic arms to maneuver around a fixed point, thus reducing chest wall pressure [21]. Moreover, in RATS, a shorter operation time and precise manipulation mitigate the adverse effects of prolonged intraoperative positioning and tissue inflammation. These ultimately result in faster pain recovery after RATS. Now, the single‐port da Vinci SP system has gradually been applied in clinical practice, and postoperative pain after RATS may be effectively relieved, potentially no worse than UVATS. Combining the recovery trends of pain and fatigue, we hypothesize that the rapid alleviation of postoperative fatigue symptoms following RATS may be associated with the faster recovery from pain, collectively contributing to the early postoperative functional recovery of patients.

Regarding additional symptoms, RATS had fewer cases of nausea and difficulty remembering in the first 2 weeks after discharge, as well as milder disturbances in insomnia and a lack of appetite, according to the EORTC QLQ‐C30 and MDASI. These advantages may be attributed equally to the delicate manipulation of RATS, which reduces the physical stress caused by surgical trauma. In addition, a shorter duration of anesthesia reduces the effects of mechanical ventilation and anesthetics on the body's internal environment and decreases the incidence of postoperative complications [37, 38].

For functional indicators, the EORTC QLQ‐C30 scale indicates that RATS performed faster recovery than UVATS in physical functioning. This subjective improvement also mirrored the objective data; RATS had a higher daily average step count than UVATS at 4 weeks after discharge, although this difference was not statistically significant. These findings suggest that RATS presents better postoperative mobility. Notably, RATS showed similar recovery trends in physical functioning, fatigue, and pain, from which we can infer that pain and fatigue directly affect patients' activity levels. Therefore, enhancing pain management, reducing interventions that cause inflammatory responses, and alleviating fatigue can improve patients' mobility and accelerate early postoperative functional recovery [39].

Existing studies on QOL have shown variable results. Jin et al. reported no significant differences in GHS/QOL between RATS and VATS at 4 weeks after discharge [40]. In contrast, Zheng et al. reported that the QOL score on the QLQ‐C30 was higher in RATS at 6 weeks after surgery [41]. In our study, RATS had a lower GHS/QoL than UVATS during the first 2 weeks after discharge on the EORTC QLQ‐C30. However, as recovery progressed, the differences between the two groups gradually narrowed. By the fourth week after discharge, both groups reached comparable recovery levels. We believe that low GHS scores in RATS are primarily related to their severe pain in the early postoperative period. Several studies have shown that pain has a significant negative impact on patients' overall health status and quality of life [42, 43]. However, within the first 4 weeks after discharge, the GHS scores in the RATS group show a faster recovery. Combined with the recovery trends of other symptoms and functional indicators, we speculate that this phenomenon may be due to the rapid alleviation of symptom burden (such as pain and fatigue) and the quick recovery of functional status (such as physical function) after discharge in RATS. However, this hypothesis still requires further validation through multi‐center, large‐sample clinical studies. Given the more favorable recovery trend in RATS, we hypothesize that with a longer follow‐up period, RATS may demonstrate superior GHS recovery compared to UVATS. These results suggest the potential long‐term benefits of RATS for postoperative functional recovery.

Although RATS has multiple advantages, its challenges and higher costs cannot be ignored. A meta‐analysis by Ma et al. revealed that the total cost of RATS is significantly greater than VATS [44]. However, our study revealed no significant difference in financial difficulties (EORTC QLQ‐C30) between the two groups, possibly because the surgical method was chosen by the patients themselves. In our hospital, RATS costs approximately $2900 USD, and it is not covered by medical insurance, so most patients have to pay for the procedure themselves, which significantly influences their decision‐making [45]. Besides, RATS lacks tactile feedback, making it difficult to sense tissue tension and texture through direct contact. This complicates controlling manipulation strength and identifying the positional relationship between the nodule and lung segment boundaries, increasing the demands on the surgeon's experience and skills and extending the learning curve.

There are several limitations of our study. First, patients were not randomized, and although PSM balanced baseline characteristics, selection bias may still exist. Second, as a single center, small sample study, the results have limited representativeness and generalizability. Third, the patients' postoperative functional recovery levels and preoperative baseline statuses were not compared. Fourth, the short follow‐up period limited the observation of long‐term functional recovery and survival. Fifth, subtle differences in the experience of surgical assistants may influence surgical outcomes. Sixth, the use of analgesic medications taken by patients on their own during the follow‐up period was not monitored, and the potential for this to affect the assessment results cannot be ruled out. Multicenter, large sample, technically standardized, high‐quality randomized clinical trials are necessary to compare, and validate the differences between these two surgical approaches in NSCLC treatment more comprehensively.

## 5 Conclusions

In the ERAS pathway, Triportal RATS generally demonstrates a comparable or even superior functional recovery trend compared to UVATS in segmentectomy, despite being associated with early pain after discharge. These findings suggest that RATS has potential advantages in early postoperative functional recovery following segmentectomy and provide a viable alternative for surgical decision‐making in early‐stage NSCLC.

## Author Contributions

All authors had full access to the data in the study and take responsibility for the integrity of the data and the accuracy of the data analysis. Conceptualization, Wang Y.C., Diao H.X., Xu L., Peng Z.M.; Data curation, Wang Y.C., Diao H.X.; Methodology, Wang Y.C., Xu L.; Investigation, Wang Y.C., Diao H.X., Xu L., Peng Z.M.; Formal Analysis, Wang Y.C., Diao H.X.; Writing – original draft preparation, Wang Y.C., Diao H.X; Writing – review and editing, Wang Y.C., Diao H.X., Xu L., Peng Z.M.; Supervision, Xu L., Peng Z.M.

## Disclosure

The authors have nothing to report.

## Conflicts of Interest

The authors declare no conflicts of interest.

## Supporting information


**Data S1.** Supporting Information.

## Data Availability

The data underlying this article will be shared on reasonable request to the corresponding author.
