# Supplementary material for: Comparison of Early Functional Recovery Following Triportal Robot‐Assisted and Uniportal Video‐Assisted Segmentectomy in Patients With Early‐Stage Non‐Small Cell Lung Cancer: A Propensity Score‐Matched Analysis
Source: Thorac Cancer. 2025 Mar 12;16(5):e70041. doi: 10.1111/1759-7714.70041 (PMC11903195; doi:10.1111/1759-7714.70041)
Supplement: Supplementary file 1 — Data S1. Supporting Information. [file TCA-16-e70041-s001.docx]

**Supplemental Material**

Table S1. Distribution of resected segments before and after propensity score-matching.

| **Tumor location** | **Before PSM (n = 135)** | | |  | **After PSM (n = 86)** | | |
| --- | --- | --- | --- | --- | --- | --- | --- |
|  | **RATS(n=45), n (%)** | **VATS(n=90), n (%)** | ***P*-Value** |  | **RATS(n=43), n (%)** | **VATS(n=43), n (%)** | ***P*-Value** |
| RS1 | 5(11.1) | 9(10.0) | 0.982 |  | 5(11.6) | 5(11.6) | 0.999 |
| RS2 | 4(8.9) | 9(10.0) |  |  | 4(9.3) | 5(11.6) |  |
| RS3 | 3(6.7) | 11(12.2) |  |  | 3(7.0) | 5(11.6) |  |
| RS6 | 3(6.7) | 9(10.0) |  |  | 3(7.0) | 3(7.0) |  |
| RS7 | 0(0.0) | 1(1.1) |  |  | 0(0.0) | 0(0.0) |  |
| RS8 | 3(6.7) | 5(5.6) |  |  | 3(7.0) | 2(4.7) |  |
| RS9 | 0(0.0) | 1(1.1) |  |  | 0(0.0) | 0(0.0) |  |
| RS10 | 2(4.4) | 1(1.1) |  |  | 1(2.3) | 1(2.3) |  |
| RS9+10 | 1(2.2) | 3(3.3) |  |  | 1(2.3) | 1(2.3) |  |
| LS1+2 | 5(11.1) | 10(11.1) |  |  | 5(11.6) | 4(9.3) |  |
| LS3 | 3(6.7) | 5(5.6) |  |  | 3(7.0) | 4(9.3) |  |
| LS1+2+3 | 1(2.2) | 5(5.6) |  |  | 1(2.3) | 1(2.3) |  |
| LS4+5 | 6(13.3) | 7(7.8) |  |  | 6(14.0) | 3(7.0) |  |
| LS6 | 5(11.1) | 9(10.0) |  |  | 5(11.6) | 5(11.6) |  |
| LS8 | 1(2.2) | 2(2.2) |  |  | 1(2.3) | 1(2.3) |  |
| LS8+9+10 | 3(6.7) | 3(3.3) |  |  | 2(4.7) | 3(7.0) |  |

Table S2. Leicester Cough Questionnaire scores (4 weeks after discharge).

|  | **After PSM (n = 86)** |  |  |
| --- | --- | --- | --- |
|  | **RATS(n=43), mean** | **UVATS(n=43), mean** | ***P*-Value** |
| **1 week** |  |  |  |
| Physical | 4.59 | 4.62 | 0.710 |
| Psychological | 4.65 | 5.05 | 0.394 |
| Social | 5.20 | 5.17 | 0.615 |
| Total scores | 14.44 | 14.84 | 0.669 |
| **2 Weeks** |  |  |  |
| Physical | 5.01 | 4.82 | 0.285 |
| Psychological | 5.20 | 5.39 | 0.473 |
| Social | 5.55 | 5.47 | 0.578 |
| Total scores | 15.77 | 15.77 | 0.782 |
| **3 Weeks** |  |  |  |
| Physical | 5.45 | 5.13 | **0.031** |
| Psychological | 5.61 | 5.57 | 0.812 |
| Social | 5.87 | 5.67 | 0.373 |
| Total scores | 17.06 | 16.35 | 0.385 |
| **4 Weeks** |  |  |  |
| Physical | 5.52 | 5.32 | 0.330 |
| Psychological | 5.74 | 5.69 | 0.856 |
| Social | 6.09 | 5.87 | 0.602 |
| Total scores | 17.34 | 16.87 | 0.769 |

Bold text hinted that these variables were statistically significant.

Table S3. MD Anderson Symptom Inventory (4 weeks after discharge).

|  | **After PSM (n = 86)** |  |  |
| --- | --- | --- | --- |
|  | **RATS(n=43)** | **UVATS(n=43)** | ***P*-Value** |
| **1 week** |  |  |  |
| Symptoms items |  |  |  |
| Pain (mean) | 5.63 | 4.49 | **0.007** |
| Fatigue (IQR) | 5(3-6) | 4(3-7) | 0.869 |
| Nausea (IQR) | 1(0-3) | 1(1-4) | **0.039** |
| Disturbed sleep (IQR) | 4(1-5) | 3(2-6) | 0.196 |
| Distress/feeling upset (IQR) | 2(1-7) | 3(1-6) | 0.458 |
| Shortness of breath (IQR) | 5(3-6) | 5(2-6) | 0.692 |
| Difficulty remembering (IQR) | 2(0-4) | 3(2-5) | **0.004** |
| Lack of appetite (IQR) | 3(2-6) | 4(2-7) | 0.258 |
| Drowsiness (IQR) | 2(2-6) | 3(2-5) | 0.739 |
| Dry mouth (IQR) | 4(1-6) | 4(2-6) | 0.642 |
| Sadness (IQR) | 2(0-5) | 2(1-4) | 0.678 |
| Vomiting (IQR) | 1(1-2) | 1(1-3) | 0.402 |
| Numbness/tingling (IQR) | 3(0-5) | 1(1-4) | 0.305 |
| Interference items |  |  |  |
| Activity (IQR) | 4(2-4) | 4(2-6) | 0.716 |
| Mood (IQR) | 2(0-5) | 3(1-5) | 0.626 |
| Working (including housework) (IQR) | 3(2-8) | 3(1-6) | 0.324 |
| Relations with other people (IQR) | 2(0-5) | 1(1-3) | 0.758 |
| Walking (IQR) | 3(2-5) | 4(2-5) | 0.643 |
| Enjoyment of life (IQR) | 3(2-4) | 3(1-5) | 0.786 |
| **2 weeks** |  |  |  |
| Symptoms items |  |  |  |
| Pain (mean) | 3.51 | 3.42 | 0.648 |
| Fatigue (IQR) | 3(3,4) | 4(3,6) | 0.235 |
| Nausea (IQR) | 1(0,2) | 1(1,3) | **0.027** |
| Disturbed sleep (IQR) | 1(0,4) | 3(1,4) | **0.020** |
| Distress/feeling upset (IQR) | 2(1,3) | 2(1,4) | 0.479 |
| Shortness of breath (IQR) | 3(3,5） | 3(2,5) | 0.954 |
| Difficulty remembering (IQR) | 2(1,3) | 3(2,4) | **0.009** |
| Lack of appetite (IQR) | 2(1,4) | 3(1,5) | 0.120 |
| Drowsiness (IQR) | 3(1,4) | 3(2,4) | 0.387 |
| Dry mouth (IQR) | 2(1,5) | 3(1,5) | 0.058 |
| Sadness (IQR) | 2(1,4) | 2(1,4) | 0.329 |
| Vomiting (IQR) | 1(1,2) | 1(1,2) | 0.396 |
| Numbness/tingling (IQR) | 1(0,5) | 2(1,3) | 0.194 |
| Interference items |  |  |  |
| Activity (IQR) | 3(1,4) | 2(1,4) | 0.752 |
| Mood (IQR) | 3(1,4) | 3(1,4) | 0.650 |
| Working (including housework) (IQR) | 4(3,5) | 3(2,5) | 0.704 |
| Relations with other people (IQR) | 2(0.4) | 2(1,3) | 0.170 |
| Walking (IQR) | 3(2,3) | 3(1,5) | 0.134 |
| Enjoyment of life (IQR) | 2(1,3) | 3(1,6) | 0.084 |
| **3 weeks** |  |  |  |
| Symptoms items |  |  |  |
| Pain (mean) | 2.98 | 3.19 | 0.543 |
| Fatigue (IQR) | 3(2,4) | 3(2,5) | **0.047** |
| Nausea (IQR) | 1(0,1) | 1(1,2) | 0.078 |
| Disturbed sleep (IQR) | 2(0,2) | 2(1,4) | **0.036** |
| Distress/feeling upset (IQR) | 2(1,3) | 2(1,4) | 0.842 |
| Shortness of breath (IQR) | 4(2,4) | 3(2,4） | 0.533 |
| Difficulty remembering (IQR) | 3(1,4) | 2(1,4) | 0.511 |
| Lack of appetite (IQR) | 2(1,3) | 3(1,4) | 0.477 |
| Drowsiness (IQR) | 2(1,3) | 3(1,4) | 0.086 |
| Dry mouth (IQR) | 2(1,4) | 3(2,5) | 0.096 |
| Sadness (IQR) | 1(1,4) | 2(1,3) | 0.164 |
| Vomiting (IQR) | 1(1,2) | 1(1,2) | 0.272 |
| Numbness/tingling (IQR) | 2(1,4) | 2(1,4） | 0.495 |
| Interference items |  |  |  |
| Activity (IQR) | 2(2,3) | 2(1,4) | 0.894 |
| Mood (IQR) | 2(1,3) | 2(1,4) | 0.316 |
| Working (including housework) (IQR) | 3(2,4) | 3(2,5) | 0.406 |
| Relations with other people (IQR) | 1(1,4) | 2(1,3) | 0.508 |
| Walking (IQR) | 3(2,4) | 2(1,5) | 0.411 |
| Enjoyment of life (IQR) | 2(1,4) | 2(1,4) | 0.361 |
| **4 weeks** |  |  |  |
| Symptoms items |  |  |  |
| Pain (mean) | 2.28 | 2.44 | 0.596 |
| Fatigue (IQR) | 2(2,3) | 3(2,4) | **0.043** |
| Nausea (IQR) | 1(1,1) | 1(1,1) | 0.179 |
| Disturbed sleep (IQR) | 2(1,2) | 2(1,3) | 0.077 |
| Distress/feeling upset (IQR) | 2(1,3) | 2(1,2) | 0.964 |
| Shortness of breath (IQR) | 3(2,5) | 2(2,3) | 0.242 |
| Difficulty remembering (IQR) | 2(1,3) | 2(1,3) | 0.432 |
| Lack of appetite (IQR) | 2(1,2) | 2(1,4) | **0.037** |
| Drowsiness (IQR) | 2(1,3) | 2(1,4) | 0.069 |
| Dry mouth (IQR) | 2(1,3) | 2(1,4) | 0.128 |
| Sadness (IQR) | 1(1,4) | 2(1,3) | 0.605 |
| Vomiting (IQR) | 1(1,1) | 1(1,2) | 0.120 |
| Numbness/tingling (IQR) | 2(1,4) | 2(1,3) | 0.565 |
| Interference items |  |  |  |
| Activity (IQR) | 2(1,3) | 2(1,3) | 0.939 |
| Mood (IQR) | 2(1,3) | 2(1,3) | 0.348 |
| Working (including housework) (IQR) | 3(2,4) | 3(2,4) | 0.619 |
| Relations with other people (IQR) | 1(1,3) | 2(1,3) | 0.174 |
| Walking (IQR) | 3(2,4) | 2(1,3) | 0.225 |
| Enjoyment of life (IQR) | 2(1,3) | 2(1,3) | 0.672 |

Bold text hinted that these variables were statistically significant.

IQR, interquartile range

Table S4. Christensen Fatigue Scale (4 weeks after discharge).

|  | **After PSM (n = 86)** |  |  |
| --- | --- | --- | --- |
|  | **RATS(n=43)** | **UVATS(n=43)** | ***P*-Value** |
| 1 week, median (IQR) | 3(3,6) | 4(3,6) | 0.056 |
| 2 weeks, median (IQR) | 3(2,4) | 4(3,6) | 0.072 |
| 3 weeks, median (IQR) | 3(2,4) | 4(2,4) | 0.166 |
| 4 weeks, median (IQR) | 2(2,3) | 3(2,4) | 0.068 |

IQR, interquartile range
